# Artificial intelligence-enhanced quantum chemical method with broad applicability

**DOI:** 10.1038/s41467-021-27340-2

**Published:** 2021-12-02

**Authors:** Peikun Zheng, Roman Zubatyuk, Wei Wu, Olexandr Isayev, Pavlo O. Dral

**Affiliations:** 1 State Key Laboratory of Physical Chemistry of Solid Surfaces, Fujian Provincial Key Laboratory of Theoretical and Computational Chemistry, Department of Chemistry, and College of Chemistry and Chemical Engineering, Xiamen University, Xiamen, 361005 China; 2 Department of Chemistry, Carnegie Mellon University, Pittsburgh, PA 15213 USA

**Keywords:** Method development, Quantum chemistry

## Abstract

High-level quantum mechanical (QM) calculations are indispensable for accurate explanation of natural phenomena on the atomistic level. Their staggering computational cost, however, poses great limitations, which luckily can be lifted to a great extent by exploiting advances in artificial intelligence (AI). Here we introduce the general-purpose, highly transferable artificial intelligence–quantum mechanical method 1 (AIQM1). It approaches the accuracy of the gold-standard coupled cluster QM method with high computational speed of the approximate low-level semiempirical QM methods for the neutral, closed-shell species in the ground state. AIQM1 can provide accurate ground-state energies for diverse organic compounds as well as geometries for even challenging systems such as large conjugated compounds (fullerene C_60_) close to experiment. This opens an opportunity to investigate chemical compounds with previously unattainable speed and accuracy as we demonstrate by determining geometries of polyyne molecules—the task difficult for both experiment and theory. Noteworthy, our method’s accuracy is also good for ions and excited-state properties, although the neural network part of AIQM1 was never fitted to these properties.

## Abstract (web summary)

Artificial intelligence is combined with quantum mechanics to break the limitations of traditional methods and create a new general-purpose method for computational chemistry simulations with high accuracy, speed and transferability.

## Introduction

Quantum mechanical (QM) methods used in chemistry are invaluable for today’s modern science as they allow insights into electronic structure at an atomistic level, which are experimentally unattainable. This in turn helps to find answers to fundamental scientific questions in chemistry and related fields, such as chemical physics and biology, and assists applied science in designing better materials and discover new medicines.

**Fig. 1:**
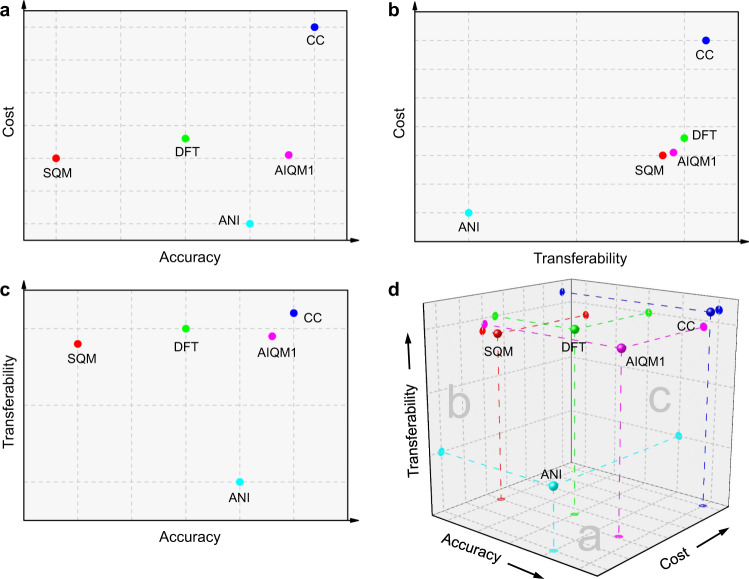
Simplified scheme of quantum chemistry approximations. Traditional quantum mechanical approaches such as the gold-standard coupled cluster (CC, blue), work-horse density functional theory (DFT, green), fast and approximate semiempirical quantum mechanical (SQM, red) methods, artificial intelligence-based ANI (cyan), and the new artificial intelligence–quantum mechanical method 1 (AIQM1, magenta). They are compared with respect to **a** cost and accuracy, **b** cost and transferability, **c** accuracy and transferability, and **d** cost, accuracy, and transferability. The accuracy of ANI is with respect to what it is applicable to. The scheme is an authors’ qualitative interpretation supported by the results of this work.

The usefulness of QM methods in practical applications is determined by their accuracy and computational cost. The trade-off between these two factors guides the choice of the QM method. On the one side, we have very accurate, but slow high-level ab initio QM methods such as coupled cluster with single, double, and perturbative triple excitations, CCSD(T)^1^, which has established itself as the gold standard in most applications, particularly, for closed-shell molecules^2–4^. On the other side, we have very fast semiempirical QM (SQM) methods that have rather limited accuracy^5^. The sweet spot of moderate computational cost and often sufficient accuracy is occupied by density functional theory (DFT) that has become a workhorse in the investigation of medium-sized systems (Fig. 1a)^6^. The efforts for developing faster and more accurate QM methods is an active research field, but it is clear that traditional approaches to QM method development require years of hard human work and typically yield only relatively modest improvements.

Advances in artificial intelligence (AI) bring chemistry research to a radically new level and provide a much-needed alternative to the traditional QM method development^7,8^. AI allows to perform calculations with both high accuracy and very low computational cost that was previously unattainable with the traditional QM methods. Nevertheless, most of the applications of AI to quantum chemistry are either proof-of-principle or limited to specific applications. Developing general-purpose AI approaches with transferability of QM methods remains a big challenge. A significant step towards transferable accurate AI approaches is the family of ANI potentials^9–12^ that can describe energies and forces of compounds of different size and composition in equilibrium and non-equilibrium configurations with accuracy approaching DFT (i.e., the ANI-1 potential trained on 20M energies of the H, C, N, and O-containing compounds at ωB97X/6-31G(d), ANI-1x trained on 5M energies at ωB97X/6-31G(d) selected by active learning, and ANI-2x extension of ANI-1x to S, F, Cl elements)^9–11^, or even coupled cluster QM level (ANI-1ccx^12^ trained on 0.5M at CCSD(T)*/CBS energies using transfer learning; CCSD(T)* is an approximation to CCSD(T) based on multi-step calculations with domain-based local-pair natural-orbital-CCSD(T)^13^ and CBS is an extrapolation to complete basis set, for the complete description of the technical details behind CCSD(T)*/CBS see refs. ^12,14^) (Fig. 1a). The ANI potentials are transferable to much larger systems than those included in the training data set, because the total energy is calculated within the local approximation by the sum of the atomic contributions with each atom feeling the environment only within some cutoff.

While impressive, ANI potentials are however much less transferable than general-purpose QM methods, because they are limited to closed-shell, neutral organic compounds, and the use of the local approximation imposes further limitations on their transferability, e.g., to large, highly conjugated systems (Fig. 1b, c). A rational approach is to exploit synergies of AI and QM methods by merging them^7^ as well as improving AI-based methods by including the effects of dispersion and long-range interactions^7,15–19^. This approach has already given rise to an increasing number of hybrid AI/QM methods^7,8,20–22^, although most of them are either proof-of-principle or based on relatively slow DFT or trained on data of limited quantity and quality potentially restricting their transferability and accuracy.

Here we describe the general-purpose artificial intelligence–quantum mechanical method 1 (AIQM1) that approaches the coupled cluster accuracy with transferability of the QM methods and computational speed of the SQM methods (Fig. 1d). AIQM1 stands on the shoulders of decades-long method development in SQM methods^5^ as well as more recent advances in developing NN potentials^15^ and combining QM with AI such as Δ-learning^23^, leveraging the power of transfer learning for exploiting limited amount of high-level reference data^12^, extensive developments in treating dispersion corrections^24,25^, efforts in accelerating high-level approaches^13^, and hard work and lots of resources invested in generating highly-accurate diverse reference data^14^. 1 in AIQM1 stands for the first iteration of the method as we envision that AIQM approaches will be further refined by using better reference data for training and making changes to the methodology, which is currently the topic of ongoing work in our labs.

## Results

### Method structure

**Fig. 2:**
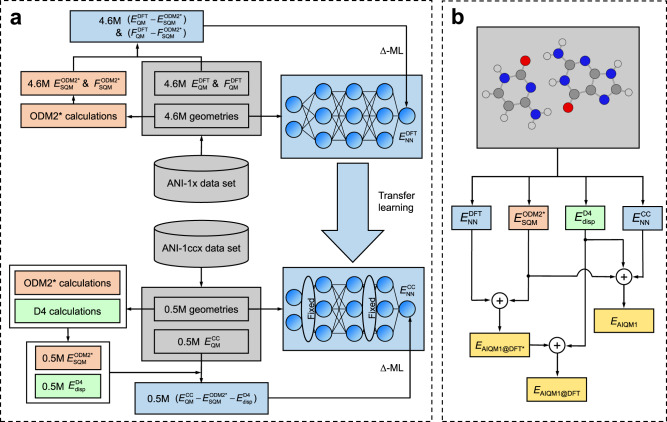
The design of the artificial intelligence–quantum mechanical method 1 (AIQM1). **a** Flowchart of training the neural network (NN) part of the AIQM1, AIQM1@DFT*, and AIQM1@DFT methods. **b** Schematic representation of the components of the AIQM1, AIQM1@DFT*, and AIQM1@DFT methods (yellow). DFT denotes density functional theory data at ωB97X/def2-TZVPP, CC— approximation to coupled cluster with single, double, and perturbative triple excitations with complete basis set extrapolation scheme (CCSD(T)*/CBS), *E* energies, *F* forces, ODM2*—semiempirical quantum mechanical (SQM) orthogonalization- and dispersion-corrected method 2 (light brown), D4—the fourth generation of dispersion (subscript disp) corrections (green), NN – neural networks of ANI-type (blue; corrections to learn also blue). Molecular data sets are in gray.

The AIQM1 method consists of three main parts (Fig. 2): (1) SQM Hamiltonian, (2) neural network (NN) correction to the potential, and (3) dispersion corrections. The AIQM1 total energy *E*_AIQM1_ is the sum of the contributions from these three parts, *E*_SQM_, *E*_NN_, *E*_disp_, respectively:

1 $${E}_{{{\mbox{AIQM}}}1}={E}_{{{\mbox{SQM}}}}+{E}_{{{\mbox{NN}}}}+{E}_{{{\mbox{disp}}}}.$$

For the first part, we have chosen the orthogonalization- and dispersion-corrected method 2 (ODM2) Hamiltonian^26^, which provides the most consistent and accurate predictions across different properties (from ground-state to excited-state and noncovalent interactions) among other SQM methods, particularly those based on neglect of diatomic differential overlap (NDDO) approximation. We remove the original D3-based dispersion corrections from the ODM2 approach and denote the modified approach as ODM2*. Instead, we add the state-of-the-art D4-dispersion corrections^24,25^ including Axilrod–Teller–Muto three-body contributions^27,28^—the third part of the AIQM1 method. Dispersion corrections are essential to properly describe dispersion terms in noncovalent interactions as they are poorly described by both SQM^5^ and local NN approaches such as ANI-1ccx^29^, and these corrections are therefore often added to AI approaches^7,15–19^. For the second part, we took the ANI-type of NN potentials. We preserved the NN-architecture of ANI-1x that predicts *E*_NN_ by summing over *N*_atoms_ atomic contributions *E*_A_:^11^

2 $${E}_{{{\mbox{NN}}}}=\mathop{\sum }\limits_{A}^{{N}_{{{\mbox{atoms}}}}}{E}_{A}.$$

We made only two minor modifications to NN model based on ANI-1x. First, we changed the activation function to GELU (Gaussian error linear unit) instead of CELU (continuously-differentiable exponential linear unit), because GELU is infinitely differentiable. This is important for applications where higher derivatives are required, e.g., geometry optimization and frequency calculations. Second, we increased the angular cutoff to 4 Å to assist with a better description of long-range interactions. Note that within ANI framework, the scalar values to learn are centered before fitting NN, i.e., the atomic contributions also include element-dependent terms obtained by linear fitting to the reference scalar values.

Our NN corrections only depend on structural parameters calculated for atoms within a cutoff. Thus, these corrections have the same limitations as the ANI models and the increased transferability of AIQM1 comes from the SQM Hamiltonian and dispersion corrections. For example, NN corrections are exactly the same for the same molecular geometry, regardless of the molecular charge or electronic state. In the future, when accurate reference data exists for diverse charged and/or electronically excited species, NN corrections can be improved by taking into account charge/electronic state as, e.g., was recently done in ref. ^30^.

### Method training and validation

We fitted NN using the ANI-1x and ANI-1ccx data sets^14^, which contain small neutral, closed-shell molecules in ground state with up to 8 non-hydrogen atoms and only considers molecules with the H, C, N, and O elements. Most of the molecules are drug-like and oligopeptides. The data sets cover not only equilibrium geometries, but also conformational space by using various sampling techniques, such as normal mode sampling and dynamics. The ANI-1x data set contains 5M geometries in total, for which ωB97X/6-31G* energies and forces were calculated (these energies were used to fit ANI-1x model). For 4.6M geometries of the ANI-1x data set, ωB97X/def2-TZVPP energies and forces are available (used previously to fit another successful general-purpose NN potential AIMNet^31^). Only energies for 0.5M geometries are available at CCSD(T)*/CBS in the ANI-1ccx data set (used to fit the ANI-1ccx potential). Such a choice of the data sets ensures high accuracy and transferability, but because of its composition, the best accuracy of AIQM1 is expected to be for ground-states energies and forces of neutral, closed-shell molecules, and it is only applicable to species with elements H, C, N, and O.

**Fig. 3:**
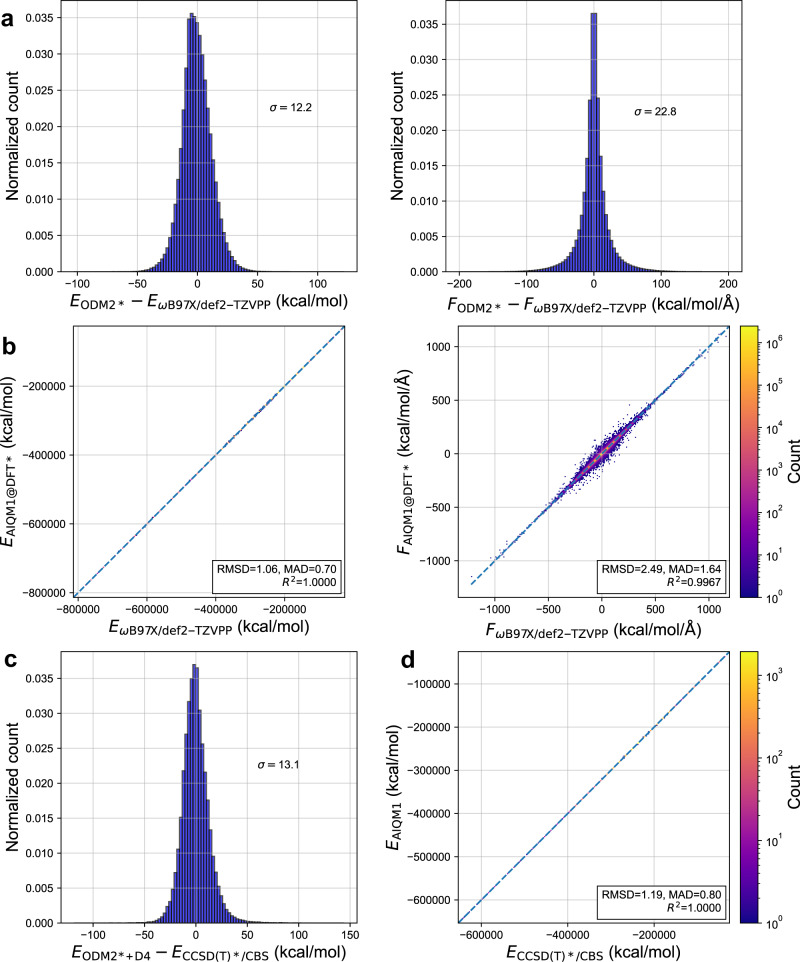
Correlation between the artificial intelligence–quantum mechanical method 1 (AIQM1) variants and reference methods for the hold-out test set. **a** Histogram of learned centered differences between ODM2* and ωB97X/def2-TZVPP energies (*E*, left) and forces (*F*, right). **b** Correlation between AIQM1@DFT* and ωB97X/def2-TZVPP energies and forces. **c** Histogram of learned centered differences between ODM2* + D4 and CCSD(T)*/CBS energies. **d** Correlation between AIQM1 and CCSD(T)*/CBS energies. Root-mean-squared deviations (RMSDs), standard deviations ($\sigma$), mean absolute deviations (MADs), and squared correlation coefficients *R*^2^ are also shown on the plots. Errors for energies are in kcal/mol, for forces—in kcal/mol/Å.

The NN weights were obtained in two steps (Fig. 2). In the first step, we fitted NN weights on the differences between the ground-state potentials calculated at DFT ωB97X/def2-TZVPP and ODM2* (see Fig. 3a for the distribution of the learned differences). This step is based on the Δ-learning^23^ approach introduced by one of us and used here to correct the low-level SQM method to the target accuracy of the higher-level DFT method with comparatively small additional computational cost. (Calculations for the entire ANI-1x data set on a single CPU are ca. 10 times faster with a single ANI-type network NN compared to SQM calculations, but the difference should become larger for bigger systems and parallel computing.) The loss function *L* in this step is the geometric mean of the loss functions for energy differences between DFT and ODM2* (*L*_E_, scalar values) and differences in forces (*L*_F_, energy gradients ${\partial {E}_{{{{{{\rm{NN}}}}}}}}/{\partial R}$ taken with opposite sign, vector values):

3 $$L=\sqrt{{L}_{E}{L}_{F}}{{{{{\boldsymbol{,}}}}}}$$

with *L*_E_ and *L*_F_ defined analogously to the loss functions for energies and forces used in ANI-2x^9^.

In this way we trained an ensemble of eight NNs (Fig. 2), which provides better accuracy than a single NN^12^ (see Methods). The method obtained in this first step is denoted by AIQM1@DFT* and it approaches DFT accuracy at the SQM cost for the hold-out test set as its mean absolute deviation (MAD) is only 0.7 kcal/mol for energies and 1.6 kcal/mol/Å for forces (Fig. 3b).

Since AIQM1@DFT* has no explicit dispersion corrections, we add the D4-dispersion corrections fitted^25^ for the DFT functional ωB97X and denote the resulting method as AIQM1@DFT.

In the second step of NN fitting (Fig. 2), we used transfer learning^32^ to reach coupled cluster accuracy using the 0.5M data points of the ANI-1ccx data set as was done for creating ANI-1ccx method^12^. Transfer learning is a powerful technique allowing to leverage more abundant training data for a related task to obtain the model for the target task using much fewer training points. For developing the AIQM1 method, we fixed the weights of the first and third hidden layers of NN from the first step to only minimize the loss function *L*_E_ for differences between the ground-state energies at CCSD(T)*/CBS and ODM2* with D4 corrections (forces are not available at CCSD(T)*/CBS and thus not included for training; see Fig. 3c for the distribution of the learned differences in energies). The resulting approach is our final AIQM1 method and it closely approaches coupled cluster level for the hold-out test set as its MAD for energies is 0.8 kcal/mol (Fig. 3d). Note that although forces and Hessians are not available at CCSD(T)*/CBS, both forces and Hessians can be easily calculated with AIQM1 with little computational cost as first- and second-order derivatives are implemented for all AIQM1 components (ODM2*, NN, and D4), which, as we will see later, is of great significance.

In the following we perform validation of our method AIQM1 and its parent variants AIQM1@DFT and AIQM1@DFT* on the independent test sets not used for fitting their NN parts. Wherever possible, we compare their performance for a range of established methods such as ODM2 (as one of the best SQM methods), B3LYP/6-31G* (because of its popularity), ωB97X/6-31G* (because it was used for generating reference data for early ANI-1 and ANI-1x models), ωB97X-D/6-31G* (as a popular representative of range-corrected DFT methods), ωB97X/def2-TZVPP (because it was used for generating reference data for AIQM1@DFT and AIQM1@DFT*), ωB97X-D4/def2-TZVPP (to test the effect of the D4 corrections), CCSD(T)*/CBS (because it was used for generating reference data for AIQM1), ANI-1ccx (best representative of the general-purpose NN potentials), and, occasionally, other relevant methods.

**Fig. 4:**
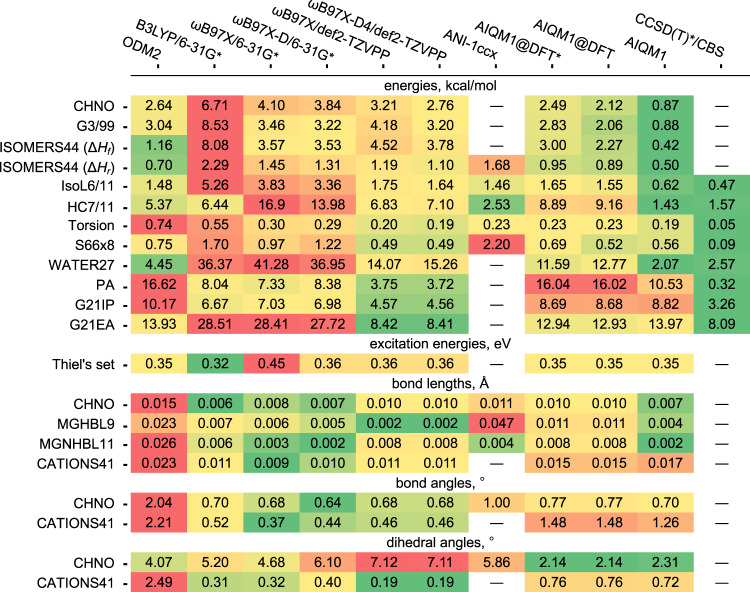
Performance of tested methods for diverse benchmarks. Mean absolute deviations (MADs) are provided for all methods and benchmark sets used in this study. MADs are color coded for easier visual analysis—red values have the highest MAD, green values—the lowest. “―” is used where (all) calculations were not possible. Data set abbreviation are their established names, i.e., CHNO—compounds with H, C, N, and O elements, G Gaussian, IsoL isomerization of larger molecules, HC hydrocarbons, IP ionization potentials, EA electron affinities, MGHBL main-group hydrogenic X–H bond lengths, MGNHBL11 main-group non-hydrogenic X–Y bond lengths; the numbers in data set names usually indicate either the original number of data points in the data set or a year it was assembled.

We cannot compare AIQM1 to ANI-1ccx for ions, radicals, and excited states, as ANI-1ccx is not transferable to such cases and they were excluded from statistics; in addition, there is no implementation for heats of formation at ANI-1ccx. No comparisons to CCSD(T)*/CBS-optimized geometries were done, because of prohibitive computational cost for such calculations and absence of implementation of analytical derivatives. This method cannot be used for excited states either. To prevent the paper from becoming unwieldy, we only focus in this text on the most important benchmarks, while the summary of calculations with all aforementioned methods can be found in Fig. 4 and Supplementary Data 1 sheet S1 and details (overview of data sets, list of compounds, reference data, and data calculated with above methods, etc.) are provided in the Supplementary Data 1 sheets S3–S20.

### Performance for energies

AIQM1 has an excellent accuracy in energies for a broad range of data sets not used for fitting its NN part (Fig. 4). A very important energy-based property is heat (enthalpy) of formation—a fundamental thermochemical quantity, which is notoriously difficult to accurately predict with quantum chemistry. Typically, only very computationally expensive QM methods are able to achieve the desired chemical accuracy for heats of formation Δ*H*_f_  (errors below 1 kcal/mol). Thus, AI was suggested as a potent approach to specifically target accurate and cost-efficient predictions of heats of formation by improving upon predictions made by the low-cost QM methods (DFT^33–35^ and SQM^36^ methods). In contrast, in our approach we did not fit NN part to better reproduce the heats of formation; we merely had to offset the bias in AIQM1 heats of formations at 298 K with respect to the experimental reference data in the CHNO data set^37^ by just fitting four parameters—atomic energies of H, C, N, and O elements, which we treat as energies of free atoms in the most stable electronic configuration at AIQM1 (Methods). The CHNO data set includes carefully curated 138 heats of formation of various molecules ranging from inorganic (H_2_, O_2_, H_2_O, NH_3_, etc.) to diverse classes of organic compounds (alkanes, alkenes, alkynes, linear and cyclic compounds, molecules with different functional groups, e.g., alcohols, amines, acids), which allowed development of general-purpose, transferable SQM methods^26,37^. This set consists of compounds with only H, C, N, and O elements, hence the name.

AIQM1 performance is remarkable for heats of formation as it easily reaches chemical accuracy for the CHNO data set (MAD of 0.9 kcal/mol), even though this property was not included in the training set of its NN part. It is the first time that a QM method with semiempirical speed has broken this threshold as, e.g., ODM2 method with the best-reported accuracy among semiempirical methods to date has three times higher MAD of 2.6 kcal/mol. Similarly, AIQM1 has MAD of 0.9 kcal/mol in heats of formation for the CHNO subset^38^ of the independent G3/99 test set (G stands for Gaussian)^39^. This subset contains species only with H, C, N, and O elements and includes 47 experimental heats of formation of medium-sized organic species (e.g., piperidine, acetal, azulene, phenyl radical etc.). The full G3/99 set formed a backbone for developing and testing many QM methods such as popular, but very computationally expensive composite approaches Gaussian-4 (G4)^40^ and G4MP2^41^ (approximation of G4 for faster calculations) targeting the coveted chemical accuracy. AIQM1 accuracy for both the CHNO and G3/99 sets is on par with G4 and G4MP2 (their MADs are in the range of 0.65–0.90 kcal/mol, see the Supplementary Data 1 sheets S4 and S5) and thus AIQM1 can be used as a computationally-efficient alternative to such composite methods. Noteworthy, AIQM1 is clearly better than DFT approaches tested here (Fig. 4).

It is important to point out the limitations of the AIQM1 as well. For example, analysis of heats of formation shows that AIQM1 has relatively large error of −2.9 kcal/mol for the H_2_ molecule, which is similar to DFT approaches (error up to 3.7 kcal/mol at ωB97X/6-31G*), but much larger than errors at G4 (−0.3 kcal/mol) and G4MP2 (−1.0 kcal/mol) (see Supplementary Data 1 sheet S4). Possible cause for such a large error is the lack of H_2_ in the ANI-1x data set used for fitting NN part of AIQM1. This example shows that AIQM1 accuracy may deteriorate significantly for cases underrepresented in its training set, regardless whether molecular structures are simple or not. On the other hand, it also shows the path to overcome such problems—by including more such cases in the training set in the future.

**Fig. 5:**
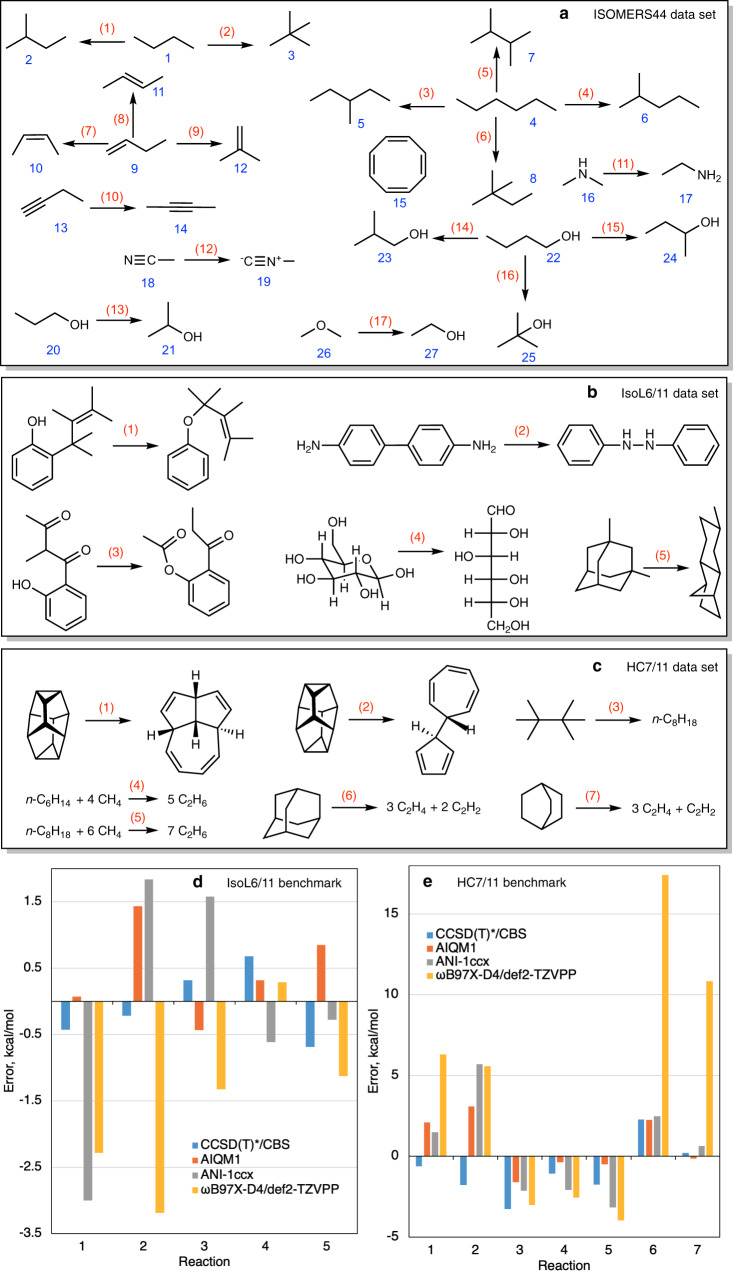
Selection of data sets for testing performance of the artificial intelligence–quantum mechanical method 1 (AIQM1) for ground-state energies. **a** ISOMERS44 set for testing accuracy in predicting heats of formation for 27 molecules (blue numbers) and 17 reaction enthalpies (red numbers), **b** reactions in IsoL6/11 set used to test accuracy in isomerization energies, **c** reactions in HC7/11 set used to test relative energies. Comparison between errors of CCSD(T)*/CBS, AIQM1, ANI-1ccx, and ωB97X-D4/def2-TZVPP for the reaction energies in the **d** IsoL6/11 and **e** HC7/11 benchmark sets.

In chemistry, we often have to deal with such relative energies as isomerization energies, reaction energies, and enthalpies as well as relative energies between conformers, because relative energies determine the outcome of reactions and 3D structures of molecules in thermal equilibrium. AIQM1 not only has good accuracy for heats of formation, but also faithfully reproduces other types of relative energies. One example is the heats of formation Δ*H*_f_ and isomerization enthalpies Δ*H*_r_ at 298 K of organic compounds in the ISOMERS44 data set^38,42^, for which AIQM1 has MAD of 0.4 and 0.5 kcal/mol, respectively. The ISOMERS44 set contains 27 experimental heats of formation of several different classes of compounds (hydrocarbons, alcohols, amines etc.) and 17 isomerization enthalpies derived from these heats of formations (Fig. 5a). The performance of AIQM1 for the ISOMERS44 set are therefore much better than performance of the DFT methods tested here (Fig. 4).

Other types of relative energies, such as zero-point energy-excluded reaction energies at 0 K are also reproduced by AIQM1 very well. For example, isomerization energies in the subset of the IsoL6/11 data set^43^ with five reactions of compounds containing only H, C, N, and O elements are reproduced by AIQM1 with chemical accuracy (MAD 0.6 kcal/mol, Fig. 5d), while the MADs of all other methods tested here are equal or larger than 1.5 kcal/mol, except for CCSD(T)*/CBS with MAD of 0.5 kcal/mol (Fig. 4). IsoL6/11 is an acronym for a data set consisting of six isomerization energies of large organic compounds. Reference energies were calculated at CCSD(T)-F12a/aug-cc-pVDZ (see Fig. 5b for isomerization reaction schemes); the whole data set is often used for testing QM methods.

Similarly, for another data set, reaction energies in the HC7/11 set^44^, AIQM1 accuracy is also very close to that^12^ of CCSD(T)*/CBS (MADs of 1.4 and 1.6 kcal/mol, respectively) and clearly outperforms all other methods having MADs from 2.5 kcal/mol (ANI-1ccx) to 16.9 kcal/mol (ωB97X/6-31G*) (Fig. 4 and Fig. 5e). HC7/11 is widely used for testing QM methods and it consists of seven difficult cases for DFT including isomerization and isodesmic energies of hydrocarbon compounds; reference energies in HC7/11 are either zero-point energy-excluded experimental values or CCSD(T)/6-311 + G(d,p) (see Fig. 5c for reaction schemes).

Relative energies of the configurations of the same molecule are also important as they define, among others, what rotational conformers are more stable, which in turn is crucial for determining 3D structures of flexible molecules. AIQM1 confidently handles this task as its median MAD for the popular torsion benchmark set^45^ is only 0.19 kcal/mol, which is the same as for much more expensive ωB97X-D4/def2-TZVPP and lower than other methods tested here (median MADs range from 0.20 to 0.74 kcal/mol, Fig. 4). We used the subset of the torsion benchmark set with only H, C, N, O-containing compounds; it consists of test cases with torsion scans for 45 fragments grouped into alkyl, aryl, aryl-amide, and bi-aryl cases with torsion profiles calculated at CCSD(T)/CBS. AIQM1 is only inferior to CCSD(T)*/CBS and MP2/CBS (median MAD 0.11 kcal/mol)^45^, which are however much slower than DFT methods tested here. Now we can turn into investigating the performance of AIQM1 for predicting geometries themselves.

### Performance for geometries

**Fig. 6:**
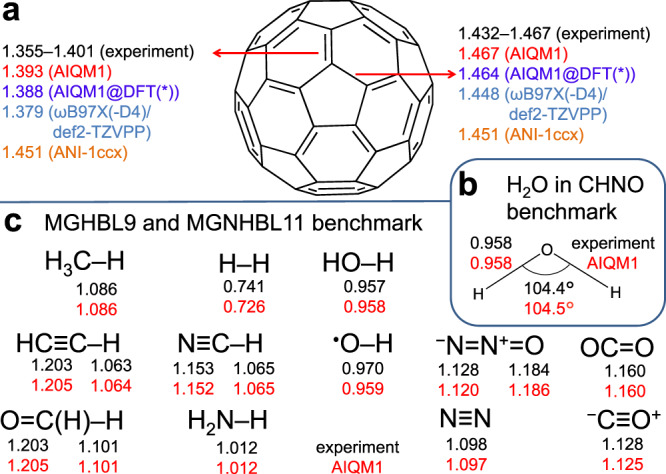
Performance of the artificial intelligence–quantum mechanical method 1 (AIQM1) for finding ground-state minimum geometries. **a** Short and long bond lengths in C_60_ as calculated at different levels of theory and compared to experimental values^46–49^. Note that geometries of C_60_ are the same at both AIQM1@DFT and AIQM1@DFT* as well as at both ωB97X and ωB97X-D4/def2-TZVPP. **b** All hydrogenic bond lengths in the MGHBL9 data set^50^ and non-hydrogenic bond lengths in the MGNHBL11 data set^50, 51^. **c** Geometry of a water molecule (one of the molecules in the CHNO benchmark; note that experimental reference is slightly different from that in MGHBL9 set in panel **b**)^37^. Bond lengths are in Å. Experimental values in black, AIQM1 values in red, other methods marked with other colors.

Theoretical prediction of molecular geometries is one of the most common applications of quantum chemistry, which is essential for chemical research as conclusive geometries are not always available from experiment. Geometry optimization is an iterative procedure requiring forces (and often Hessians), which makes it much more computationally expensive than energy calculations for a single geometry. SQM methods are much less accurate for geometries than common DFT methods and general-purpose NN potentials fail to deal with subtle conjugation effects, e.g., ANI-1ccx predicts that all bond lengths in C_60_ are equal to 1.451 Å, while it is known from experiment^46–49^ that bond length between two adjacent hexagon rings is shorter than bond length between pentagon and hexagon rings (Fig. 6a).

Optimization with AIQM1 forces successfully distinguishes these two bond types in C_60_ and predicts short and long bond lengths to be 1.393 and 1.467 Å, respectively (Fig. 6a). For this molecule, we cannot compare AIQM1 predictions with CCSD(T)*/CBS due to the staggering cost of this coupled cluster approach (single-point energy calculations take 69 hours on 15 CPU cores), while experimental data are not conclusive as they range from 1.355 to 1.401 Å for short bond length and from 1.432 to 1.467 Å for long bond length depending on measurement conditions^46–49^. Instead, we compare AIQM1@DFT predicting 1.388 and 1.464 Å to ωB97X-D4/def2-TZVPP predictions of 1.379 and 1.449 Å, which are in acceptable agreement (Fig. 6a), while the cost of geometry optimization with AIQM1@DFT* is 14 s on a single CPU core vs. 31 min on 32 CPU cores at DFT.

For smaller molecules, where reliable data is available, AIQM1 has very good accuracy, much better than, e.g., the accuracy of ODM2 or ANI-1ccx. AIQM1 is also more consistent than DFT methods, whose performance strongly depends on the functional and basis set (Fig. 4). For the CHNO data set^37^ with experimental reference data, the MADs of AIQM1, ODM2, and ANI-1ccx are 0.007, 0.015, and 0.011 Å in bond lengths, 0.70°, 2.04°, and 1.00° in bond angles, and 2.31°, 4.07°, and 5.86° in dihedral angles, respectively (see, e.g., excellent prediction of water geometry, Fig. 6b). Similarly, for nine main-group hydrogenic X–H bond lengths (MGHBL9)^50^ and 9 main-group non-hydrogenic X–Y bond lengths (MGNHBL11)^50,51^ data sets with experimental data used to test DFT methods, MAD of AIQM1 in bond lengths is 0.004 and 0.002 Å (Fig. 6c), respectively, which is again much better than ODM2 (0.023 and 0.026 Å) or ANI-1ccx (0.047 and 0.004 Å). The latter two data sets contain small molecules H_2_, CH_4_, NH_3_, H_2_O, HF, C_2_H_2_, HCN, H_2_CO, CO, N_2_, F_2_, CO_2_, N_2_O, and OH radical (investigated bond lengths are shown in Fig. 6c; OH radical was excluded from statistics of ANI-1ccx). We only excluded molecules Cl_2_ and MgS from the full MGNHBL11 data set.

### Predicting polyyne structures

**Fig. 7:**
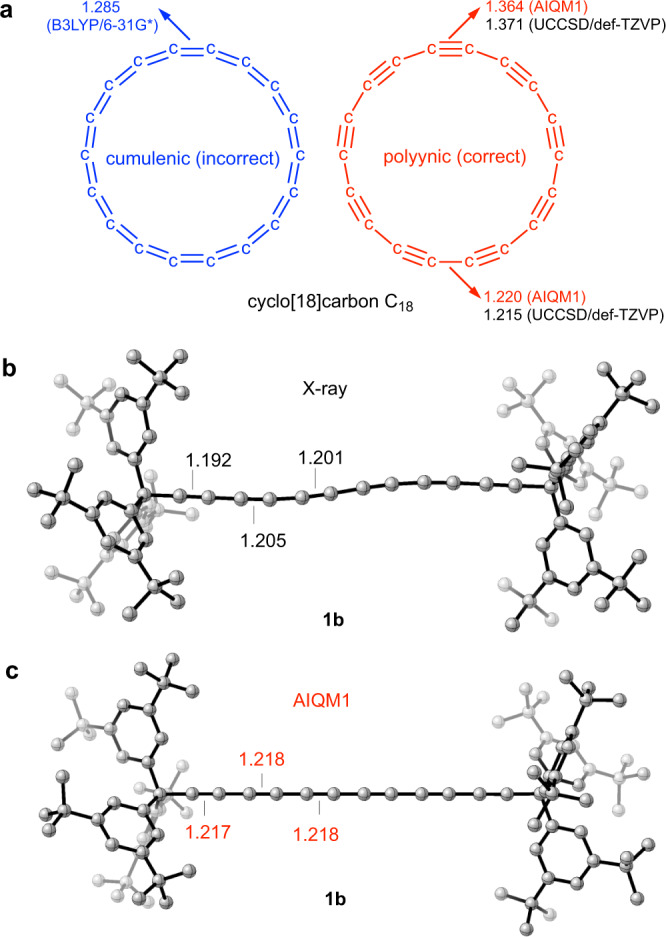
Geometries of polyyne compounds. **a** Correct polyynic (red) geometry of C_18_ predicted by AIQM1 (bond lengths in red) and UCCSD/def-TZVP (bond lengths in black from ref. ^56^) and its incorrect alternative cumulenic (blue) geometry predicted by B3LYP/6-31G* (bond lengths in blue). **b** X-ray structure of **1b** compound from ref. ^57^ assigning too short triple C≡C bond lengths (shown in black). **c** AIQM1 geometry of **1b** with revised bond lengths (shown in red). Bond lengths are in Å. Other bond lengths and the coordinates of AIQM1 geometry are given in the Supplementary Data 1 sheet S23.

AIQM1 opens the door for calculating geometries with previously unattainable accuracy and speed, which is crucial for compounds, whose structural determination is difficult both experimentally and theoretically. One such case is cyclo[18]carbon C_18_, which was inspiring the imagination of chemists from 1966^52^, but whose accurate geometrical parameters are still unknown despite many efforts by both experimentalists and theoreticians^53–56^. Experiment only shows that this molecule has polyynic structure with alternating bond lengths rather than cumulenic structure with equal bond lengths^55^ (Fig. 7a). C_18_ is extremely challenging for QM methods too: some DFT methods predict wrong cumulenic structure (Fig. 7a), while others correct polyynic structure (Fig. 7)^53–56^. The best theoretical estimates reported to date are at coupled cluster with single and double excitations (CCSD), which neglect important triple excitations and use rather small basis sets (due to the very high computational cost of CCSD geometry optimizations, the largest basis set used was only def-TZVP)^53,56^. We optimized C_18_ at AIQM1 without imposing any symmetry constraints in contrast to previous theoretical works (where such constraints were also necessary to reduce the computational cost) and report the revised best theoretical estimate of the geometry with short bond lengths of 1.220 Å and long bond lengths of 1.364  Å. These calculations only took 2 seconds on a single CPU. In retrospect, previous unrestricted CCSD (UCCSD/def-TZVP) calculations^56^ (bong lengths of 1.215 Å and long bond length of 1.371 Å, Fig. 7a), are much closer to the AIQM1 result than DFT approaches benchmarked previously^56^ against UCCSD/def-TZVP (e.g., the best DFT method was found^56^ to be ωB97X-D/def2-TZVP predicting 1.221  Å and 1.344  Å).

As a further demonstration of AIQM1 capabilities for polyynes, we report a structure of a free molecule of a model polyyne **1b** from ref. ^57^. It has six C≡C bonds, but whopping 224 atoms due to bulky tris(3,5-di-t-butylphenyl)methyl (Tr*) end groups used for protection. As we have previously shown^58^, electronic properties such as optical band gap of this molecule strongly depend on its geometry and therefore accurate optimizations of this class of compounds are of high importance. Optimization with coupled cluster methods is at the moment impossible due to their prohibitive cost for such a large number of atoms, while the truncation of structure will not fully capture the effect of the end groups. X-ray structure of **1b** is available^57^; nevertheless, it is well known^59–61^ that the triple C≡C bond length determined by X-ray diffraction experiments are severely shortened due to high electron density in the middle of these bonds. AIQM1 revises the lengths of the triple C≡C bonds to be 0.013–0.025 Å longer than in previously reported X-ray structures (Fig. 7b, c). In addition, X-ray structures are significantly impacted by packing and vibrational effects depending on temperature and the measured structures have pronounced S-shaped bend, while a free standing **1b** molecule in vacuum is predicted by AIQM1 to be linear.

We hope that the future studies on these polyyne molecules with better experimental and theoretical methods can provide a conclusive, independent validation of our predictions with AIQM1. As indirect validation of AIQM1 serves its low MAD of 0.004  Å in seven triple C≡C bond lengths present in the CHNO set (see also Fig. 6c for an example of excellent accuracy of AIQM1 for the acetylene molecule in the MGNHBL11 set).

### Performance for noncovalent interactions

**Fig. 8:**
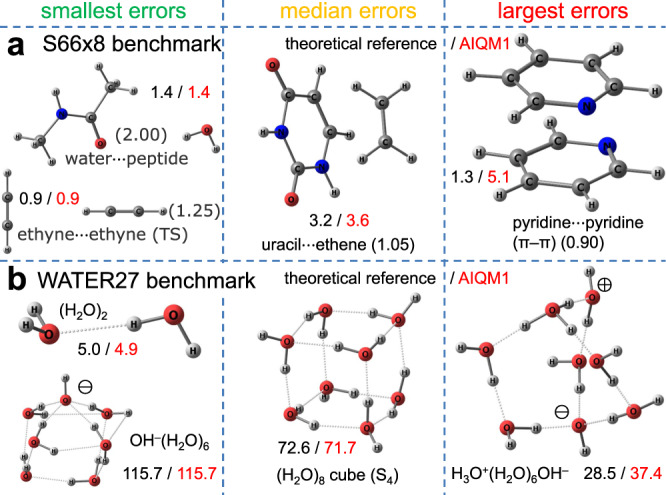
Performance of the artificial intelligence–quantum mechanical method 1 (AIQM1) for noncovalent interactions. Selection of complexes with errors ranging from smallest to median to largest values for **a** the S66x8 benchmark and **b** the WATER27 benchmark. In the figure, interaction energies are shown in kcal/mol, black at reference and red at AIQM1. Other numbers such as in “(1.25) ethyne⋅⋅⋅ethyne (TS)” correspond to the scaling of the distance between monomers relative to equilibrium structures as used for construction of the S66x8 set.

AIQM1 is transferable to noncovalent interactions, which are very challenging even for the state-of-the-art QM methods and NN potentials. For the standard benchmark set S66x8 with CCSD(T)/CBS reference noncovalent interaction energies^62^, AIQM1 has rather good accuracy as its MAD is 0.6 kcal/mol, which is comparable to ODM2 (0.8 kcal/mol) and DFT, e.g., ωB97X-D/6-31G* (1.2 kcal/mol) and ωB97X-D4/def2-TZVPP (0.5 kcal/mol) (see Fig. 4 for MADs and Fig. 8 for selected structures). S66x8 set contains 66 noncovalent complexes in their equilibrium geometries and geometries with displaced monomers (in total 528 geometries) and it represents different types of interaction (electrostatic- and dispersion-dominated as well as mixed types). Hence, AIQM1 is a good cost-efficient alternative to many DFT methods.

The method performance is particularly good for hydrogen-bonded complexes. For 27 clusters of neutral water molecules (H_2_O)_n_, and charged clusters H^+^(H_2_O)_n_ and OH^−^(H_2_O)_n_ (WATER27 data set^63^ with revised values^64^ for (H_2_O)_20_ clusters), AIQM1 has MAD of only 2.1 kcal/mol compared to 4.5 of ODM2 (see Fig. 4 for MADs and Fig. 8 for selected structures). This makes the method competitive in terms of accuracy with popular dispersion-corrected DFT approaches, which have similar errors (see, e.g., ref. ^64^), but are much slower. AIQM1 is therefore a promising method for simulating chemical processes in water solutions, essential for biological processes. It is noteworthy that this data set contains charged species, which can be adequately described neither by ANI-1ccx nor by the DFT methods tested here as the basis sets are not adequate for treating anionic species, which brings us to the next topic.

### Beyond closed-shell, neutral molecules

AIQM1 is transferable beyond closed-shell, neutral species used for fitting its NN part and even improves upon the ODM2 method (ANI potentials cannot be used at all for such simulations). We saw before that AIQM1 performs well for charged protonated and deprotonated water clusters. Other examples are proton affinities, where MAD is improved from 16.6 (ODM2) to 10.5 (AIQM1) kcal/mol for the proton affinities (PA) data set^63^ and MAD in adiabatic ionization potentials (G21IP set)^63^ from 10.2 to 8.8 kcal/mol (Fig. 4). Nevertheless, MAD in adiabatic electron affinities (G21EA set)^63^ is practically the same for both ODM2 and AIQM1 (ca. 14.0 kcal/mol). All these data sets consist of experimental reference values for small compounds, and here we used only their subset with species containing at least two atoms and only H, C, N, O elements: PA has eight proton affinities of H_2_, H_2_O, NH_3_, and five unsaturated hydrocarbons, IP21 and EA13 both have nine (albeit not the same) small organic and inorganic species (see Supplementary Data 1 sheets S13–S15 for the list of species, reference, and calculated data). In general, DFT outperforms AIQM1 (Fig. 4) for the benchmarked cationic species (PA and G21IP sets), but DFT performance has strong dependence on the basis set and, e.g., calculations with 6-31G* have similar or even larger errors than AIQM1, especially after removing the biggest outlier in AIQM1, which is the proton affinity of the H_2_ molecule underestimated by −35.4 kcal/mol (see the Supplementary Data 1 sheet S13).

Anionic species (G21EA set) are even more difficult and require large, diffuse basis sets for proper QM treatment as is clear by comparing MAD of DFT approaches, which ranges from ca. 28 kcal/mol with the 6-31G* basis set to 8.4 kcal/mol with larger def2-TZVPP basis set; even CCSD(T)*/CBS has a large error of 8.09 kcal/mol (Fig. 4). Thus, a rather large error of AIQM1 (14.0 kcal/mol) is not surprising and the proper treatment of electron affinities remains a big challenge to be addressed in the future.

Interestingly, geometries are also improved for charged species as for the CATIONS41 data set^38,65^, the MADs of AIQM1 and ODM2 are 0.017 and 0.023 Å in bond lengths, 1.26° and 2.21° in bond angles, and 0.72° and 2.49° in dihedral angles, respectively. The CATIONS41 data set consists of 75 bond lengths, 38 bond angles, and five dihedral angles, determined experimentally and by using high-level theoretical methods, of small organic (CH^+^, C_2_H_3_^+^, C_2_H_5_^+^, propargyl cation, cyclopropenyl cation etc.) and inorganic (triplet and singlet OH^+^, NO^+^, NH_4_^+^ etc.) species. Tested cations are, however, better described by DFT (Fig. 4) than by AIQM1.

All in all, there is clearly a room for improvement of AIQM1 method for ionic species. Nevertheless, all the tests were performed here for rather small molecules, for which reliable reference data is available, while in case of larger systems, where the charge is more delocalized, AIQM1 is expected to perform better as the electronic density will be more similar to the corresponding neutral species.

### Beyond ground-state properties

**Fig. 9:**
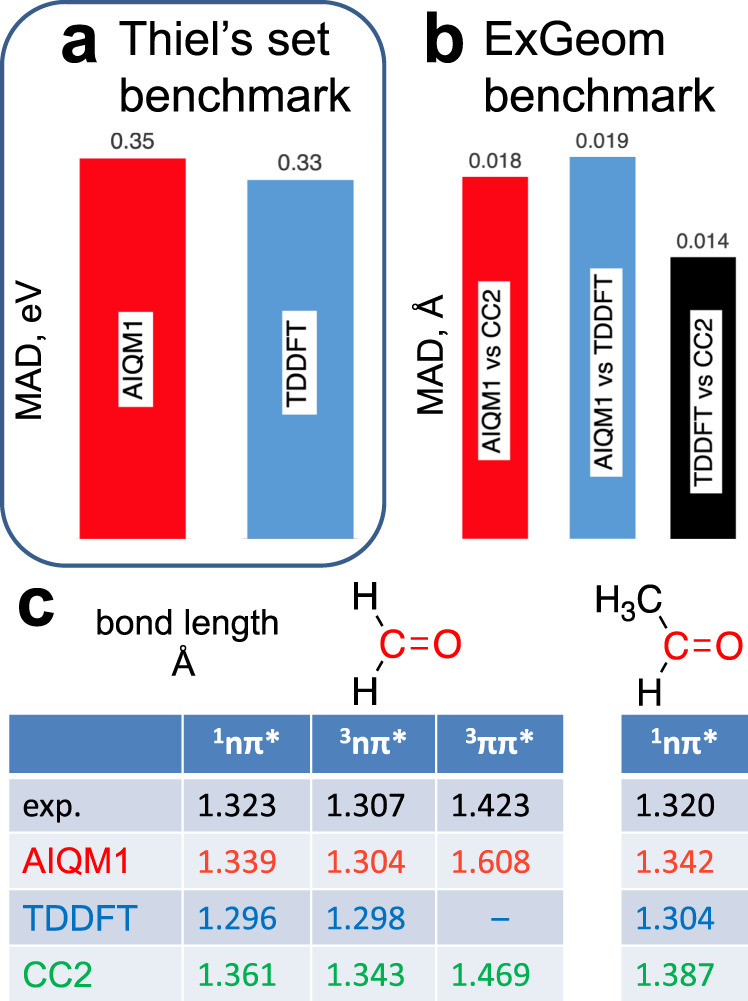
Performance of the artificial intelligence–quantum mechanical method 1 (AIQM1) for excited states. **a** Mean absolute error (MAD) in vertical excitation energies for Thiel’s benchmark set^66^ (AIQM1 in red, time-dependent density functional theory, TDDFT, in blue). **b** MAD in bond lengths for the ExGeom benchmark set^26, 66^ (AIQM1 vs approximate coupled cluster singles-and-doubles method (CC2) in red, AIQM1 vs. TDDFT in blue, TDDFT vs. CC2 in black). **c** Lengths of C–O bonds in formaldehyde and acetaldehyde as compared to experiment (experiment in black, AIQM1 in red, TDDFT in blue, CC2 in green; one value for TDDFT is missing due to the failed geometry optimization at this level^66^). Values for TDDFT (B3LYP/TZVP), CC2, TDDFT vs. CC2, and experiment are taken from ref. ^66^.

Finally, AIQM1 method is also transferable to electronically excited states and, e.g., it can be used for multi-reference configuration interaction (MRCI) calculations to predict excitation energies, oscillator strengths and nonadiabatic couplings for simulating spectra and performing nonadiabatic excited-state dynamics. Here we use the graphical unitary-group approach (GUGA) and the same settings (active space, excitation levels, etc.) for MRCI calculations as previously used for benchmarking SQM methods (see Methods for details)^26,66,67^. AIQM1/MRCI is three orders of magnitude faster than popular linear-response time-dependent (TD) DFT approaches such as TD-B3LYP, TD-ωB97X, and TD-ωB97X-D, while the accuracy in vertical excitation energies is similar for these methods (MAD of AIQM1/MRCI is 0.35 eV, which is close to TD-DFT methods with MAD of 0.32–0.45 eV for the Thiel’s data set^66,67^, Fig. 4 and Fig. 9a). Thiel’s set is often used for benchmarking QM methods and consists of 167 singlet and triplet vertical excitation energies for several states of 28 middle-sized organic compounds represented by unsaturated linear and cyclic hydrocarbons as well as heterocycles calculated with multistate multiconfigurational second-order perturbation theory (MS-CASPT2/aug-cc-pVTZ) for most compounds and with equation-of-motion (EOM)-CCSD(T)/aug-cc-pVTZ for nucleobases cytosine, thymine, and adenine.

MRCI calculations are performed using the SQM (ODM2*) Hamiltonian of AIQM1 and thus, excitation energies are trivially the same as in ODM2* and ODM2. However, when calculating forces for molecules in excited states, NN corrections to forces are added and their effect is not clear as they were trained on ground-state reference data. Thus, we test AIQM1/MRCI forces, by performing geometry optimizations of molecules in excited-states. Such optimizations are of large importance and required, e.g., for simulating fluorescence spectra, but they are very computationally expensive with QM methods and thus the low-cost of AIQM1 makes it potentially attractive for this task. We tested AIQM1/MRCI performance on the ExGeom set^26,66^ with excited-state geometries and AIQM1/MRCI MAD for bond lengths is 0.018 Å vs. the approximate coupled cluster singles-and-doubles method (CC2) reference (with TZVP basis set) and 0.019 Å vs. TDDFT reference (specifically, TD-B3LYP/TZVP). This is rather good result given that uncertainties of the reference calculations are in the same order of magnitude (MAD of TDDFT reference vs. CC2 reference is 0.014 Å, Fig. 9b)^66^. The ExGeom set consists of more than 500 reference C–C, C–H, C–O, C–N, and N–H bond lengths of 32 molecules of different classes (e.g., aldehydes, ketones, nucleobases, heterocycles) in different excited states with altogether 100 excited-state equilibrium geometries. Accurate experimental values are very hard to obtain. However, for the available experimental bond lengths in the ExGeom data set, AIQM1/MRCI gives better or similar predictions compared to TDDFT and CC2 for C–O bond in ^1^nπ* and ^3^nπ* excited states, while its error is much bigger for the ^3^ππ* excited state of formaldehyde (Fig. 9c).

Overall, AIQM1 seems to be a better choice than current routinely used QM methods in terms of performance/cost ratio at least for some types of excitations, which holds a great promise for using this method for exploration of dynamical properties arising from the manifold of electronic states, e.g., by performing nonadiabatic excited-state dynamics, which should be an interesting topic for future explorations. In any case, the AIQM1 method is only the first step in the direction of creating a general-purpose AI-based method for excited-state simulations—an important, but open topic in chemistry^68^—as obviously training models on excited-state properties will be crucial for future improvements.

## Discussion

After initial excitement about great promises AI holds for substituting QM methods, the focus is shifting towards tighter integration of AI with QM instead of substituting QM altogether. This shift is motivated by the need to incorporate correct physical behavior of QM methods, while at the same time exploiting great ability of AI to improve low-level QM methods’ accuracy without compromising their speed.

In this work, we have made a step towards creating general-purpose AI-improved QM methods useful for a variety of applications out-of-the-box. Our approach AIQM1 synergistically combines the best of two worlds—transferability of QM and high accuracy of AI approaches. The success of this approach only became possible with great advances over recent years in methodology development of both QM and AI components as well as generation of numerous carefully curated, high quality reference data. Thus, AIQM1 allows very accurate prediction of ground-state properties such as energies and geometries of closed-shell, neutral organic compounds approaching the gold-standard CCSD(T)/CBS at the speed of semiempirical QM methods. Remarkably, it has accuracy improved in comparison to the parent SQM method (ODM2) also for other cases, not explicitly considered during training of its NN part, e.g., for charged species, showcasing the benefits of using physically-motivated AI. Thus, AIQM1 method has the potential to become a very useful tool for routine simulations with high accuracy.

It is only the beginning of the exciting road for AI-improved QM methods for general-purpose applications. In the near future we expect tighter integration of AI with QM, further optimizing both AI and QM parts, training on more and higher quality reference data, and further extending transferability and accuracy for all properties of interest to chemists and physicists.

## Methods

### Neural network training

The neural network training and evaluation was performed with the TorchANI software^69^. Each NN-part of AIQM1@DFT* consists of an ensemble of eight ANI-type NNs, which provides better accuracy according to our tests. The ensemble was trained similarly to the previous procedure^12^, i.e., the data set was split into nine equal parts, with one part held out for testing and the remaining eight parts were used as cross-validation splits for training eight networks. Each network was trained on seven cross-validation splits and validated on one split using standard rotation of splits. During the training of AIQM1@DFT*, we stopped training NN after 1000 epochs, because we found that longer training does not improve much the performance for the validation set, but deteriorates performance for some of the external data sets. When we analyzed the error between AIQM1@DFT* predicted values and reference DFT values, we found several outliers with error >0.01 a.u. By recalculating the DFT values for these outliers, we found their reference values in ANI-1x data set were wrong, so we used the updated values to train our models. Transfer learning was then used to refit above eight ANI-type networks to 80% of the entire set with CCSD(T)*/CBS values to obtain the final NN part of AIQM1 consisting of ensemble of eight NNs; other 10% were used as the validation set and remaining 10% as the hold-out test set.

### Calculation of enthalpies

The enthalpies at 298 K were calculated within harmonic oscillator and rigid rotor approximation in our locally modified version of the MNDO program^70^. Calculating heats of formation requires the evaluation of the atomization energies, which depend on the choice of the atomic energies. Atomic energies calculated with CCSD(T)*/CBS used for fitting NN-part of AIQM1 lead to large errors in atomization energies even for moderate-sized molecules such as naphthalene (error of 25.4 kcal/mol with respect to CCSD(T)/CBS, where the two-point extrapolation scheme was used with cc-pVDZ and cc-pVTZ basis sets); thus we fitted atomic energies of H, C, N, and O elements to reduce the error in heats of formation in the CHNO set. Heats of formation calculated at AIQM1@DFT and AIQM1@DFT* use atomic energies calculated with ωB97X/def2-TZVPP. All values of atomic energies are reported in the Supplementary Data 1 sheet S2.

Heats of formation at other levels (G4, G4MP2, DFT) were calculated using a standard procedure^71^. The procedure for calculating heats of formation with the MNDO program for ODM2, AIQM1, AIQM1@DFT*, and AIQM1@DFT is equivalent, but directly uses experimental reference values for heats of formation of atomic species at 298 K^5^, which are slightly different than those used in G4, G4MP2, and DFT.

### Electronic structure and benchmark calculations

All ODM2 and ODM2* calculations were carried out with the MNDO program^70^. CCSD(T)*/CBS calculations were performed with the ORCA 4.2.0 software package^72,73^ following the procedure defined in literature^12^. The ωB97X-D4 calculations were performed with ORCA 4.2.0, and ωB97X-D calculations were performed with Gaussian 16^74^. The ωB97X/6-31G* calculations were performed with Gaussian 16, while ωB97X/def2-TZVPP calculations were performed with ORCA 4.2.0. D4-dispersion corrections were calculated with the dftd4 program^75^. We performed benchmarks of AIQM1, AIQM1@DFT*, and AIQM1@DFT with the locally modified version of the MNDO program^70^ interfaced to TorchANI^69^ and dftd4^75^. For benchmarking excited-state properties with AIQM1/MRCI, we used the same settings (active spaces, excitation levels, etc.) as in MRCI calculations with ODM2/MRCI^26,66,67^. Specifically, for benchmarking vertical excitations in the Thiel’s set, we used the single-reference CISDTQ, which closely approximates full CI and is more accurate than, e.g., MR-CISD approximation to full CI; the active spaces include all π molecular orbitals for *π* → π* excitations and also include lone-pair molecular orbitals for *n* → π* excitations; the starting reference electronic configuration is the ground-state SCF determinant. For benchmarking excited-state geometry optimizations with MRCI, we used MR-CISD level in most cases as well as MR-CISDT and MR-CISDTQ in a few cases; the active spaces and reference electronic configurations are the same as provided in the Supporting Information of ref. ^66^. All the data for benchmarks can be found in the Supplementary Data 1. The Supplementary Data 2 with Cartesian coordinates for the CHNO, CATIONS41, and ExGeom data sets is also provided.

### Geometry optimizations

The ωB97X/def2-TZVPP, ωB97X-D4/def2-TZVPP geometry optimizations were performed with the ORCA program using the default BFGS algorithm, while B3LYP/6-31G*, ωB97X/6-31G*, ωB97X-D/6-31G* geometry optimizations were performed with Gaussian 16 using the default Berny algorithm GEDIIS. For ANI-1ccx and AIQM1, the geometry optimizations are performed by interfacing to the MNDO program using the default BFGS algorithm for most data sets except for optimizations of C_60_, C_18_, **1b** (optimized by interfacing to Gaussian 16) and the torsion benchmark (optimized by interfacing to ASE^76^ using the LBFGS algorithm).

## Supplementary information

Description of Additional Supplementary Files

Supplementary Data 1

Supplementary Data 2

## Data Availability

The data (calculated energies and optimized geometries) generated in this study are provided in the Supplementary Information. Any other relevant data are available from the authors upon reasonable request.

## Code availability

The AIQM1, AIQM1@DFT, and AIQM1@DFT* methods are available as the open-source code free of charge for non-commercial and non-profit uses, such as academic research and education, as described in http://MLatom.com/AIQM1. Any other relevant code is available from the authors upon reasonable request.
