# Supplementary material for: Artificial intelligence-enhanced quantum chemical method with broad applicability
Source: Nat Commun. 2021 Dec 2;12:7022. doi: 10.1038/s41467-021-27340-2 (PMC8640006; doi:10.1038/s41467-021-27340-2)
Supplement: Supplementary file 1 — Description of Additional Supplementary Files [file 41467_2021_27340_MOESM1_ESM.pdf]

Description of Additional Supplementary Files

File Name: Supplementary Data 1
Description: Excel file with the atomic energies to calculate AIQM1 enthalpies of formation; raw data with reference energies and energies calculated for the article for benchmarks of energies.

File Name: Supplementary Data 2
Description: zip archive file with Cartesian coordinates for species in the CHNO, CATIONS41, and ExGeom data sets used for benchmarks of geometries.
